# Impact of Pollutant Ozone on the Biophysical Properties of Tear Film Lipid Layer Model Membranes

**DOI:** 10.3390/membranes13020165

**Published:** 2023-01-28

**Authors:** Mahshid Keramatnejad, Christine DeWolf

**Affiliations:** Department of Chemistry and Biochemistry and Centre for NanoScience Research, Concordia University, Montreal, QC H4B 1R6, Canada

**Keywords:** tear film model membranes, cholesteryl oleate, glyceryl trioleate, Brewster angle microscopy, Langmuir film, dilational rheology, mass spectrometry, ozone, environmental smog, dry eye disease

## Abstract

Ozone exposure from environmental smog has been implicated as a risk factor for developing dry eye disease (DED). The tear film lipid layer (TFLL), which is the outermost layer of the tear film and responsible for surface tension reduction while blinking, is in direct contact with the environment and serves as the first line of defense against external aggressors such as environmental pollution. The impact of exposure to ozone on the biophysical properties of three TFLL model membranes was investigated. These model membranes include a binary mixture of cholesteryl oleate (CO) and L-α-phosphatidylcholine (egg PC), a ternary mixture of CO, glyceryl trioleate (GT) and PC, as well as a quaternary mixture of CO, GT, a mixture of free fatty acids palmitic acid and stearic acid (FFAs) and PC. Biophysical impacts were evaluated as changes to the surface activity, respreadability, morphology and viscoelastic properties of the films. Expansion to higher molecular areas was observed in all the TFLL model membrane films which is attributable to the accommodation of the cleaved chains in the film. Significant morphological changes were observed, namely fluidization and the disruption of the phase transition behaviour of GT, and multilayer formation of CO. This fluidization reduces the hysteresis loops for the model membranes. On the other hand, the viscoelastic properties of the films exhibited differential impacts from ozone exposure as a function of composition. These findings are correlated to chemical changes to the lipids determined using ESI-MS.

## 1. Introduction

The tear film lipid layer (TFLL) is the final layer of the human tear film which covers the surface of the cornea and is responsible for creating the air–tear interface [1]. The TFLL is mainly composed of lipids secreted by the meibomian glands [2,3]. It has been reported that the composition of the TFLL is >90% non-polar lipids [1,2,4,5] with a smaller percentage of polar lipids. The meibum is composed of 30–45 mol% cholesteryl esters (CEs), 30–50 mol% wax esters [1,2,3,4,5,6,7] 2~4 mol% triglycerides [2,4,5,8], 0.5–5.6 mol% free cholesterol [2,4,5,8,9,10,11], 2 mol% diesters [4,9,10,11] and 3–7 mol% hydrocarbons [4,9,10,11]. The polar lipids of the TFLL are reported to be 3–5 mol% (O-Acyl)-ω-hydroxy fatty acids (OAHFA) [3,12,13], ~13 mol% phospholipids [1,2,14] and up to 2.5 mol% free fatty acids [4,9,10,11], of meibomian and non-meibomian origins.

Despite the controversies in the literature and the challenges regarding the determination of the exact composition of the TFLL, there is no doubt that this 15–160 nm-thick interface is paramount in the function of blinking, responsible for tear surface tension reduction [1,15] and the stability of the tear film while blinking by respreading after each blink, due to its distinct surface characteristics [1,2,16]. The application of structural techniques such as grazing incidence Xray diffraction (GIXD) has provided information on the two-dimensional phase order of the TFLL as well as its layered organization [17,18]. Thus, a multilayered structure is suggested for the TFLL [19,20,21], with the polar lipids creating an amphiphilic sublayer on top of the aqueous tear film, and the non-polar lipids creating a hydrophobic bulky layer on top of the polar amphiphilic layer [1,2,9,22]. The amphiphilic nature of the polar sub-layer aids in the spreading of the non-polar lipids on the aqueous layer underneath, thus allowing the stabilization of the TFLL [1,2,9,16,21,23].

Disruption in the composition and structure of TFLL leads to the development of dry eye disease (DED), which is a multifactorial disease impacting the ocular surface [24]. Meibomian gland dysfunction (MGD) is reported in 86% of DED cases [24,25], wherein an unstable and patchy tear film as well as a non-invasive break up time of <5 s are reported compared with the stable and continuous tear film in healthy subjects, which has a non-invasive break up time of >15 s [2,26,27,28,29]. DED is one of the major ophthalmic health concerns of the modern world [2,24], with a life quality altering effect on 10–30% of the global population [30] and an estimated annual cost of $55.4 billion [2,31] in the USA, and a similar global impact [2,32]. It is characterized by instability in the tear film, inflammation in the ocular surface and tear hyperosmolarity [2,24]. With a prevalence of 21.3% in Canada [33], many risk factors have been associated with DED, including MGD [2,34], age [30,35,36,37], race [24], sex [24,30,35,37,38] and most recently, air pollution [30,37,39,40,41].

Many studies in the recent years have reported a correlation between ophthalmic surface diseases and air pollution, with a special emphasis on ozone. Air pollution was reported as one of the risk factors of DED in a study on USA veterans [42,43]. In a time-stratified study in in Hangzhou, China, higher concentrations of air pollutants were significantly associated with DED [39]. A smog analysis in Lahore associated increases in the concentrations of pollutants NO_x_, PM_10_, O_3,_ SO_2_, CO, VOC as well as PM_2.5_ with an overall 60% increase in the number of ocular surface patients, with dry eye, irritation and lid erosion being the major contributors [40]. Significant associations have been found between DED prevalence and increased ozone levels in Korea [41,44]. Among other air pollutants, O_3_ was reported as a DED risk factor in a large-scale study in China wherein the prevalence of DED was found to be 61.57% [37]. Moreover, exposure to ozone long term is associated with increase in inflammation, lower tear production and lower conjunctival goblet cell density [45,46].

Tropospheric ozone is produced from the photolysis reaction of NO_2_ in the troposphere layer, which is produced the reaction of NO with the hydroperoxyl radicals (HO_2_) that are produced from the reaction of hydrocarbons with hydroxyl radicals [47,48,49,50]. An increase in fossil fuel and biomass burning has caused an increase in surface levels of NO_x_ radicals [47,49,51] and has led to a major reported increase in ground levels of ozone compared to the pre-industrial era, from 10 to 15 ppb then to 30 to 40 ppb now [49,51], with levels as high as 300 ppb in very polluted areas, and photochemical smog [52,53,54].

**Figure 1:**
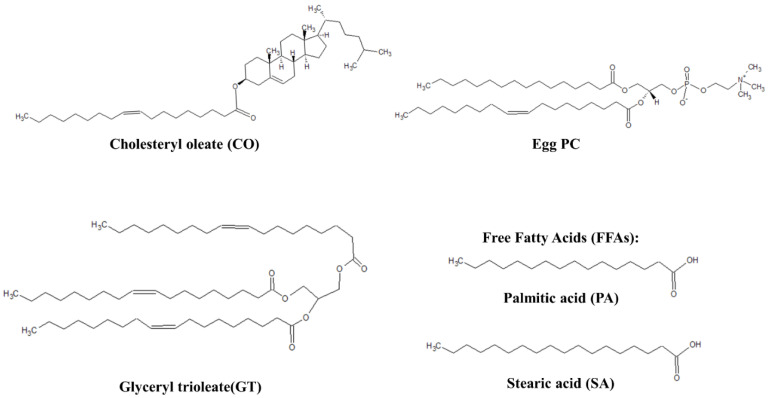
Representative lipids of TFLL model membranes.

Despite the many studies focused on the correlation between DED prevalence and ground-level ozone concentrations, the research on the impact of ozone exposure on the mechanical properties and biophysical characteristics of the TFLL is limited. Ozone is capable of reacting with the unsaturated double bond in the acyl chain of TFLL components via the Criegee mechanism, as was shown in a study focused on the effect of ambient ozone exposure on wax esters [55]. Thus, with a large proportion of the TFLL components’ acyl chains containing one or more unsaturated carbon-carbon double bonds [8], it is valuable to understand the chemical and physical impact of ozone oxidation on the TFLL. In this work, the impact of ozone exposure on the biophysical and surface characteristics of three TFLL model membranes is investigated (Figure 1), specifically binary, ternary and quaternary mixtures composed of key components (cholesteryl oleate, glyceryl trioleate, L-α-phosphatidylcholine and free fatty acids) and a physiologically relevant non-polar:polar ratio. The surface activity of the model membranes, as well as cycle experiments to study the respreadability of the films, are studied using a Langmuir balance. Brewster angle microscopy (BAM) is used to study the morphology of the films, and the dilational rheology experiments were carried out using pendant drop experiments by a profile analysis tensiometer (PAT). The outcomes are correlated with the presence of ozonolysis products determined by ESI-MS on ozone-exposed films.

## 2. Materials and Methods

### 2.1. Materials

L-α-phosphatidylcholine (egg PC, >99%) was purchased from Avanti Polar Lipids (Oakville, ON, Canada). Cholesteryl oleate (CO, >98%), glyceryl trioleate (GT, ≥99%), palmitic acid (PA, >99%), stearic acid (SA, >99%) and phosphate-buffered saline tablets (PBS, pH 7.4, 10 mM phosphate, 137 mM NaCl, 2.7 mM KCl) were purchased from Sigma-Aldrich (Oakville, ON, Canada). HPLC grade chloroform was purchased from Fisher Scientific (Saint-Laurent, QC, Canada) and used in all experiments as the spreading solvent.

### 2.2. Preparation of Mixtures, Solutions and Subphases

Stock solutions of CO, Egg PC and GT were used to reach a molar ratio of 90:10 for the binary mixture and 40:40:20 for the ternary mixture. Stock solutions of PA and SA were used to achieve a molar ratio of 50:50 for the FFA (free fatty acids) mixture to be used as a component of the quaternary mixture. Solutions of CO, GT, FFA and Egg PC were used to reach molar ratios of 40:25:15:20, respectively and make the CO:GT:FFA:PC (quaternary mixture) mixture. The PBS buffer subphase was prepared by dissolving one phosphate-buffered saline tablet in 200 mL of ultrapure water with a resistivity of 18.2 MΩ cm^−1^ from a Milli-Q^®^ HX 7080 (HC) water purification system (Millipore Sigma, Oakville, ON, Canada).

### 2.3. Langmuir Film Balance

A Langmuir film balance (NIMA Technologies, Coventry, UK, 170 cm^2^) was used to obtain surface pressure-area isotherm on which the surface pressure (π) is obtained using a filter paper Wilhelmy plate. Monolayer solutions (~1.0 mg mL^−1^) were spread on PBS buffer on a Langmuir trough and 10 min allowed for the films to equilibrate and the chloroform solvent to evaporate. After the equilibration time, the barriers of the Langmuir balance closed at a speed of 5 cm^2^ min^−1^ to compress the films and produce the surface pressure-area isotherms. For the compression–expansion cycles, the films were compressed and expanded for 6 consecutive cycles at a speed of (196 cm^2^ min^−1^), which is the highest achievable speed with this Langmuir trough. At least 3 reproducible measurements were performed for each system.

### 2.4. Ozone Exposure

The Langmuir trough was enclosed with a Plexiglass cover equipped with an inlet and outlet at opposite ends. After spreading the films, and after the 10-min equilibration time, the films were exposed to ozone concentrations of an average of 800 ppb, with a flow rate of 100 mL min^−1^ for a period of 30 min. To generate ozone, first, dry and hydrocarbon-free air was generated by passing compressed air first through a drying tube filled with anhydrous calcium sulfate and then a VOC scrubber and a ChromGas zero air generator (Parker, Milton, ON, Canada), before passing through a UVP (Upland, CA, USA) ozone generator with a Pen-Ray lamp (wavelength 185 nm, power 2 to 20 watts). The ozone concentration was determined based on the absorption level of UV light at 254 nm using a 2B Technology ozone monitor, and the gas flow rate was monitored with an Aalborg digital mass flow controller.

### 2.5. Brewster Angle Microscopy

Brewster angle microscopy (BAM) was performed through coupling Langmuir film balance from NIMA Technologies (Coventry, UK) with an I-Elli2000 imaging ellipsometer (I-Elli2000, Nanofilm Technologies, Goettingen, Germany). This ellipsometer has a 50 mW Nd:YAG laser (λ = 532 nm). Model membranes were spread and compressed as described above, and images were obtained during compression with a 20X magnification lens, a lateral resolution of 1 µm, and an incident angle of 53.15°. For ozone exposed films, the plexiglass cover was removed prior to imaging.

### 2.6. Profile Analysis Tensiometry

A profile analysis tensiometer (PAT) from SINTERFACE (Berlin Germany) was used to obtain rheological parameters (film dilational viscosity and elasticity); a detailed description of this method can be found in the literature [56,57,58]. Chloroform solutions with lipid concentrations up to 0.1 mg mL^−1^ were used to spread films on the surface of a PBS pendant drop with a volume of 13 µL and an area of 32 mm^2^. After an equilibration time of 3 min, the area was increased to 40 mm^2^ followed by an additional 3 min of equilibration. After the equilibration time, a pre-programmed set of molecular area steps (described below) was used to perform rheological measurements on the films at different surface pressures along the isotherm.

Axisymmetric drop shape analysis (ASDA) [59,60,61] is used to relate the drop curvature to the surface pressure using the Young–Laplace equation [59,62]. ADSA allows determination of the monolayer surface tension (σ), pressure (π), dilatational surface elasticity (the real component of the complex modulus) and dilatational surface viscosity (derived from the imaginary part of the complex modulus). [63,64]. The drop profile is captured by a camera, and it is important for the drop to retain Laplacian shape throughout the experiments. Thus, there must be negligible disturbance of the drop profile (e.g., through harmonic distortions) and drop loss while the measurements are being performed [59]. This can be achieved by correctly choosing the oscillation frequency and the amplitude of the rheological measurements, as it has been previously reported that they can impact rheological measurements [59,60,65,66,67]. An amplitude of 2.50% (of the drop area) was chosen in order to minimize such effects [59]. The drop is oscillated at a constant frequency, and 0.16 Hz was used as it represents the frequency of blinking (i.e., 10 blinks per minute in normal subjects, as reported previously in the literature) [68]. For each model membrane, a stepwise program was designed to account for the changes in the slope of the surface pressure-area isotherms of each model membrane film. This ensures that sufficient measurement points are taken to ensure maximum surface pressure coverage (each 1 mN min^−1^ change in the surface pressure). The details of the programs used for each system are provided in the Supplementary Material. For each system, at least 4 separate measurements (i.e., on independently formed films) were obtained.

Ozone exposure experiments were performed by first spreading the film, followed by a 3-min equilibration time, after which the drop was exposed to ozone with the same parameters as above in a 40 mL reaction chamber prior to each rheology measurement.

### 2.7. Mass Spectrometry

Electrospray ionization mass spectrometry (ESI-MS) was performed on a Thermo LTQ Orbitrap Velos mass spectrometer (Markham, ON, Canada) in both negative and positive ESI modes to identify the ozonolysis products. MS samples were prepared by spreading the film on a pendant drop and exposing it to an average of 800 ppb ozone for a period of 30 min with similar parameters as the rheology ozone-exposure experiment. An average of 6 to 10 drops were collected to reach sufficient material for analysis (approximately 30 ng). The drops containing the oxidized lipid material were dried under nitrogen, resolubilized in a 2:1 chloroform/methanol solution and directly injected in the ESI-MS using an autosampler. Full scans were performed in an m/z range of 15–2000. Mass spectra were analyzed using the software FreeStyle ^TM^ 1.8 SP2. A putative identification of products was performed based on MS1 data only, by comparison to the theoretical mass of known products and pathways.

## 3. Results

### 3.1. Surface Activity and Morphology of TFLL Model Membranes

**Figure 2:**
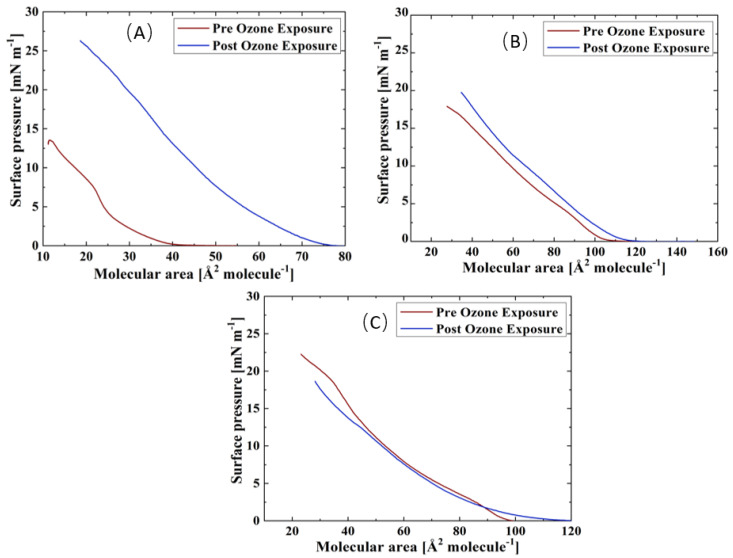
Isotherms of (**A**) CO:PC; (**B**) CO:GT:PC (top, right); and (**C**) CO:GT:FFA:PC (bottom) before ozone exposure (red) and after ozone exposure (blue) on PBS at 22 °C.

Surface pressure-area isotherms of the three TFLL model membrane systems are presented in Figure 2. The isotherms and morphology of unoxidized films were reported previously and will not be discussed in detail here [69]. For all three systems, an expansion to higher molecular areas is observed in their ozone exposed isotherms, which is the most pronounced in the case of the binary CO:PC mixture, and less so consecutively for the ternary CO:GT:PC and quaternary CO:GT:FFA:PC mixtures. Similar behaviour was observed in previous work in ozone-exposed lung surfactant model membranes composed of phospholipids DPPC and POPG, where only POPG contains an unsaturated acyl chain in its structure and can be oxidised by ozone [47,59,70].

It is known that ozone reacts with the carbon-carbon double bond via the Criegee reaction mechanism [71,72,73], wherein the initial ozonide formed leads to the formation of a Criegee intermediate and generates final major products of aldehydes, hydroxy hydroperoxides or carboxylic acids [74,75,76]. For phospholipids, these products include both water-insoluble lipid derivatives and water-soluble, short-chain species (e.g., nonanal nonanoic acid) [77]. In the case of the ozonolysis of wax esters by ambient ozone, in addition to these products, a stable ozonide has been observed [55]. Specifically considering the TFLL components utilized in this work, CO, GT, and egg PC have unsaturation in their acyl chains and are predisposed to oxidation by ozone (for the egg PC > 53% of the fatty acid distribution comprises unsaturated chains, and the majority of these lipid components comprise at least one unsaturated chain). Thus, the expansion observed in the isotherms could be due to accommodation within the film of the cleaved lipid chains which are the products of the ozonolysis and have altered surface characteristics. Most notably, given the increased polarity upon chain cleavage, the oxidized chains will disrupt the film organization and disturb the packing of the lipid molecules. These alterations affect film permeability and enable the water molecules to enter the film [59,73].

Interestingly, despite the oxidation, all three films retain their amphiphilic characteristics and surface activity, displaying similar or higher surface pressures as they are compressed to lower molecular areas. Moreover, oxidation has also impacted the phase-change behaviour of the films, i.e., a smoothing effect can be observed in their compression isotherms, where previously distinct phase transitions would be observed; this will be further discussed using BAM imaging.

Following exposure to ozone, the lipid films were collected for analysis by ESI-MS, which confirmed the existence of the aldehydes, carboxylic acids, hydroxy hydroperoxides, and stable ozonide as well as, interestingly, some Criegee intermediates, all of which contribute to the film’s net surface behaviour (Supplemental Information Tables S1–S3). With respect to the latter, to the best of our knowledge, Criegee intermediates in the context of a surface reaction have not been directly observed in MS analysis. It was previously reported by Thompson et al. 2013 [70] that the exposure of 1-palmitoyl-2-oleoyl-sn-glycero-3-phosphocholine (POPC) to ozone leads to the C9 (nonanal/nonanoic acid) fragment being lost, either entering the bulk phase or gas phase, and the increase in the surface pressure was attributed not to the incorporation of the separated C9 fragment into the film but to the rearrangement of the molecules to allow for the reversal in the orientation of the damaged oxidized acyl chain, and its incorporation into the film led by the formation of an aldehyde or carboxylic acid group at the tail [47,70].

Compared with the ternary and the quaternary mixture films, the increase in surface activity for the binary CO:PC mixture is much more prominent (an almost two-fold increase). Since 90% of the composition of this mixture is CO, it is reasonable to assume that with nonanal leaving the film through either evaporation or dissolution, the enhanced surface activity observed in the binary film post oxidation may be due to the incorporation of the CO aldehyde- and carboxylic acid-terminated chains into the film through orientation reversal or the stable ozonide remaining in the film, in addition to the aldehyde and carboxylic species derived from egg PC oxidation [47,70].

As noted already, the ternary CO:GT:PC mixture also shows an expansion to higher molecular areas post oxidation that is less extensive than that observed with the binary mixture. ESI-MS revealed that oxidation of GT yielded products with varying degrees of reaction progress, that is, combinations of unreacted chains, Criegee intermediates, fully oxidized aldehydes and carboxylic acids (Supplemental Information Table S2). In some products, all of the chains are found in the fully reacted aldehyde form; moreover, no stable ozonide is observed. These products may also be retained at the interface. Considering the ESI-MS shows oxidation of the unsaturated acyl chains of CO, GT and PC, it is likely that there are competing factors (incorporation versus dissolution of products) that lead to the smaller impact of oxidation on the ternary mixture isotherm.

The quaternary CO:GT:FFA:PC mixture is less predisposed to oxidation due to the saturated acyl chains of the FFA which comprise 15% of the film which yields the smaller film expansion. The small reduction in surface activity at low molecular areas in the oxidized quaternary film is attributed to the increased proportion of GT (and hence GT-oxidation products) in this model membrane. As the reduction of surface tension is paramount as one of the major functions of the TFLL, the reduction in the maximum surface pressure attained by the film due to the existence of damaged lipid species in the film will strain the overall TFLL system. Moreover, the oxidation has affected the envelope transition of GT [78,79] in both the ternary and quaternary films (as evidenced both in the isotherms and BAM images, to be discussed below). This could imply that the oxidized GT species do not have a typical envelope phase transition.

**Figure 3:**
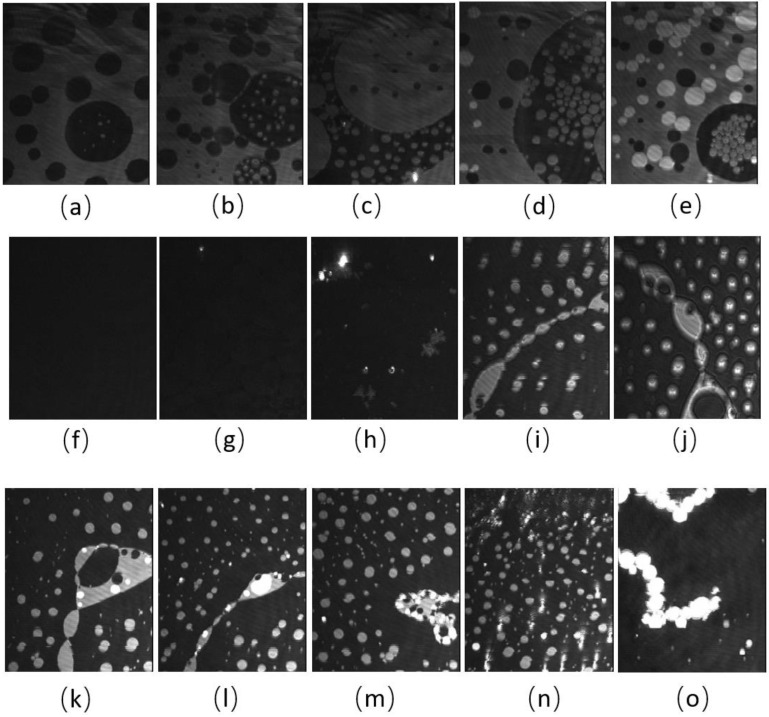
(**a**–**j**) BAM images (220 µm wide) of CO:PC 90:10 (binary mixture) film before ozone exposure (**a**–**e**) and after ozone exposure (**f**–**j**) at surface pressures of 2 mNm^−1^, 5 mNm^−1^, 7 mNm^−1^, 13 mNm^−1^ and 14 mNm^−1^ on PBS at 22 °C. (**k**–**o**) BAM images (220 µm wide) of CO:PC 90:10 (binary mixture) film after ozone exposure at surface pressures of 16 mNm^−1^, 18 mNm^−1^, 19 mNm^−1^, 23 mNm^−1^ and 20 mNm^−1^ on PBS at 22 °C (the last image was taken as the pressure decreases after compression is stopped).

BAM images of the CO:PC 90:10 film (Figure 3) show significant morphological changes to the film after ozone oxidation. The oxidized film shows what appears to be a predominantly gaseous or LE phase. Enhancing the brightness and contrast of the post-oxidation images reveals the appearance of circular domains with very low contrast (Supplemental Information, Figure S2), whereas at the same pressures for the unoxidized film, the growth of the higher contrast condensed phase domains could be easily observed. These low contrast domains coalesce by 7 mNm^−1^, and irregular aggregates with higher contrast appear (Figure 3c, 7 mNm^−1^). In agreement with the isotherm shift to higher molecular areas, the oxidation has led to a predominantly monolayer film. This is in stark contrast to the unoxidized film, where at the same pressures, a large surface percentage of the film is predominantly covered by condensed phase domains and multilayers. Thus, the oxidation of CO unsaturated acyl chains impacts both the nature of the phases formed and hinders/limits the eventual multilayering, which is observed on the unoxidized film at higher surface pressures. More regular and brighter domains only begin to appear from 12 mNm^−1^ in the ozone-exposed binary film, although with much lower density than the unoxidized film, which at the same pressures already shows a predominantly condensed phase with evidence of multilayering (two brightness levels). For the oxidized film, much less multilayering is observed; this is only at higher surface pressures (Figure 3k,l,m,n,o), surface pressures that are not achievable with the unoxidized film. Notably, once the barriers have stopped compressing, the surface pressure almost immediately decreases and large areas of very bright, chain-like aggregates are detected (Figure 3o); this is evidence that the oxidation also disrupts the otherwise very stable film characteristics.

**Figure 4:**
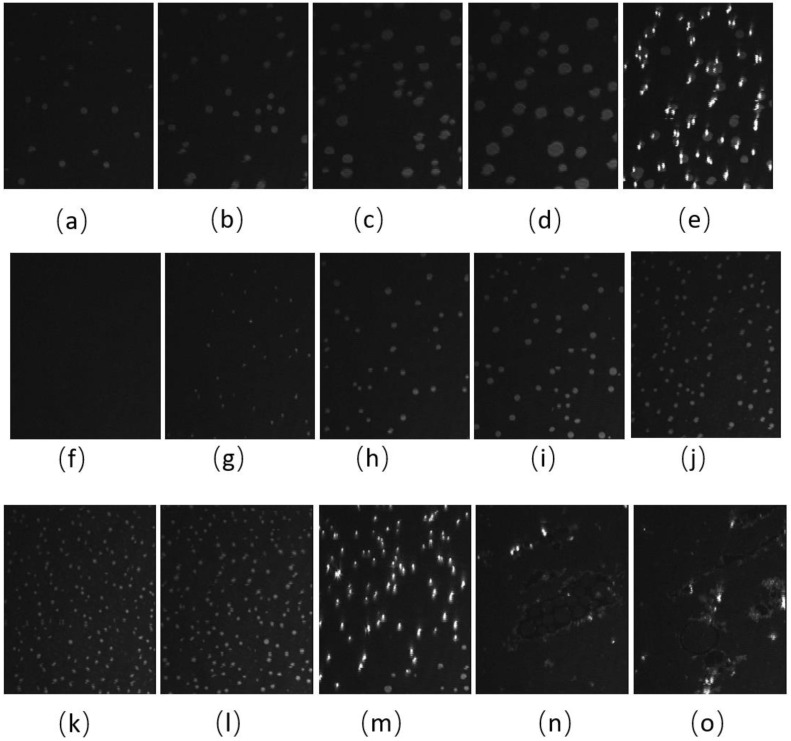
(**a**–**j**) BAM images (220 µm wide) of CO:GT:PC 40:40:20 (ternary mixture) film before ozone exposure (**a**–**e**) and after ozone exposure (**f**–**j**) at surface pressures of 7 mNm^−1^, 9 mNm^−1^, 12 mNm^−1^, 14 mNm^−1^ and, 17 mNm^−1^ on PBS at 22 °C. (**k**,**l**) BAM images (220 µm wide) of CO:GT:PC 40:40:20 (ternary mixture) film at surface pressures of 18 mNm^−1^ and 20 mNm^−1^ after ozone exposure on PBS at 22 °C. (**m**–**o**) BAM images of CO:GT:PC 40:40:20 (ternary mixture) film at surface pressures (left to right) of 18 mNm^−1^, 16 mNm^−1^, 15 mNm^−1^ after ozone exposure, taken after the end of film compression.

The BAM images of CO:GT:PC 40:40:20 (ternary mixture) (Figure 4) also show fluidization upon oxidation, with the formation of much smaller CO domains than the unoxidized film and less area coverage by the condensed phase. With the oxidation of the CO acyl chain, the domains remain smaller throughout the compression. Most notable is the impact of oxidation on the envelope transition of GT. With the unoxidized film, the transition of GT into aggregates of similar diameter occurs through the expulsion of GT from the film, as was previously observed [78], whereas this transition into three-dimensional aggregates is no longer observable with the oxidized film; this may be attributed to one or more of the GT acyl chains being oxidized. Instead, the oxidized film shows small domains of CO increasing in number, with little growth in size, as the surface pressure increases again to surface pressures higher than those achievable with the unoxidized film. Similar to the binary mixture, immediately after the compression is complete and the Langmuir trough barriers remain closed (Figure 4m,n,o), the surface pressure of the film sharply decreases, and the film shows the formation of clusters of more irregularly shaped domains closely associated with brighter (higher) 3-dimensional aggregates.

**Figure 5:**
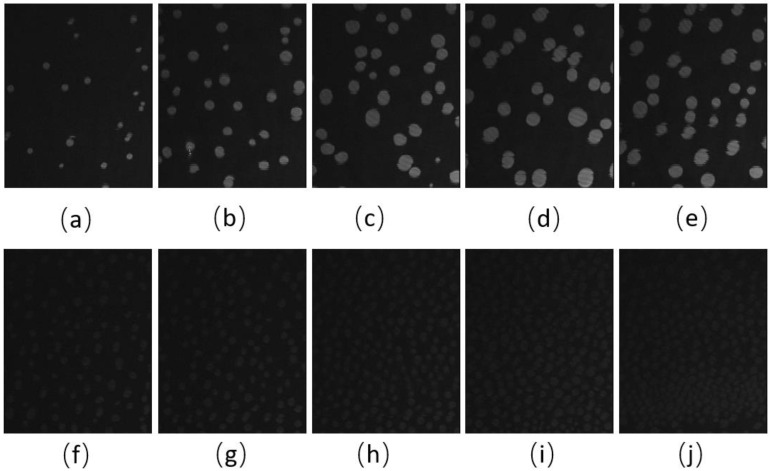
(**a**–**j**) BAM images (220 µm wide) of CO:GT:FFA:PC 40:25:15:20 (quaternary mixture) film before ozone exposure (**a**–**e**) and after ozone exposure (**f**–**j**) at surface pressures of 5 mNm^−1^, 9 mNm^−1^, 13 mNm^−1^, 16 mNm^−1^ and, 18 mNm^−1^ on PBS at 22 °C.

The BAM images of the CO:GT:FFA:PC 40:25:15:20 (quaternary mixture) (Figure 5 and Figure S2) show that for the same surface pressures, there is a higher density of CO condensed phase domains with the unoxidized film, although again the domain contrast with the surrounding phase is diminished, which may be due to a decrease in domain thickness due to chain cleavage (brightness- and contrast-enhanced, post-oxidation images are found in Figure S2). While the unoxidized films show a growth in domain size with compression, this is not the case post-ozone exposure, although the surface coverage and density of the CO domains continues to increase. Despite the lack of oxidation in FFA chains, the smaller size of the CO domains on the oxidized film may still be due to the presence of the saturated chain FFAs which affect the line tension and are enriched in these domains as other components are oxidized. A similar impact was previously observed as a result of PA on a lung surfactant model membrane [47].

### 3.2. Compression-Expansion Cycles of TFLL Model Membranes

**Figure 6:**
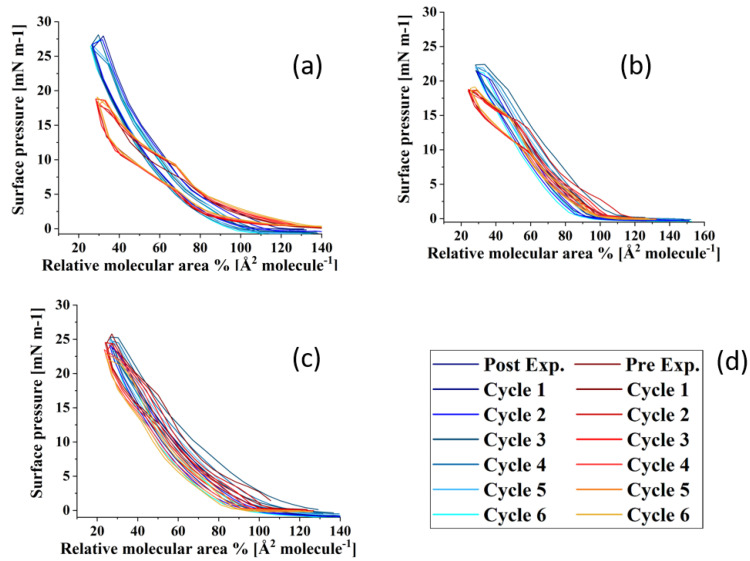
Compression–expansion cycles in terms of relative area of (**a**) CO:PC 90:10 (binary mixture); (**b**) CO:GT:PC 40:40:20 (ternary mixture); and (**c**) CO:GT:FFA:PC 40:25:15:20 (quaternary mixture, bottom) before ozone exposure ((**d**), red color family) and after ozone exposure ((**d**), blue color family) on PBS at 22 °C.

**Figure 7:**
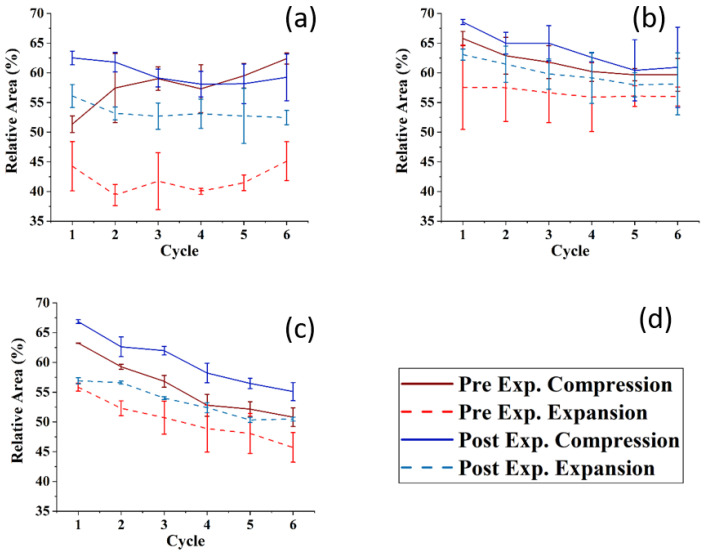
The relative area at 10 mNm^−1^ of the compression ((**d**), dark red line: pre-ozone exposure, dark blue line: post-ozone exposure) and expansion ((**d**), dashed light red: pre-ozone exposure, dashed light blue: post-ozone exposure) as a function of the cycle number for (**a**) CO:PC 90:10 (binary mixture); (**b**) CO:GT:PC 40:40:20 (ternary mixture); and (**c**) CO:GT:FFA:PC 40:25:15:20 (quaternary mixture) on PBS at 22 °C. The data points and error bars represent the mean and standard deviation of at least three independent cycling experiments.

Figure 6 shows the compression–expansion cycles of the model TFLL films pre- and post exposure to ozone. The compression–expansion rate used (196 cm^2^ min^−1^) is slower than both the average blinking rate of 10 blinks per minute [68] and the rate at which the eyelid closes [80]; however, it is the highest speed possible with the Langmuir trough used. Figure 7 shows the change in relative area percentage at a representative pressure of 10 mN m^−1^ as a function of cycle number. The relative area percentage is the percentage of the film area relative to the molecular area of the first cycle measured at 2 mN m^−1^. The compression–expansion cycles shown in Figure 6 have been normalized to their relative area for easier comparison between systems (the cycles as a function of their molecular area are provided in the Supplemental Information, Figure S3). There are two key parameters that are evaluated through these compression–expansion cycles, namely: (1) changes to the hysteresis loop, in which the area between the compression and expansion represents the work completed in the cycle representing the blinking process; and (2) the extent to which the films recover after each subsequent cycle, that is, the shift in area per molecule, which is indicated by the slope of the change in the relative area % as a function of cycle number. High respreadability and reversibility are of paramount importance in the proper function of a healthy TFLL [16,80] and are observed typically as a characteristic of extracted meibum films [81,82,83,84].

For the CO:PC film, despite the expansion observed due to oxidation and the increased surface activity (Figure 2), the impact of oxidation on the respreadability of the films is fairly small, which is not unexpected given the fluidization of the film and the relatively high respreadability pre-oxidation which has previously been reported as a consequence of reversible multilayer formation. The hysteresis loop, assessed as the difference between the relative areas for the compression and expansion segments of the loop at the same surface pressure (Figure 7), is significantly reduced with the oxidized film. However, it should be noted that the loss of the distinct phase transition amplifies the difference at this particular pressure (10 mNm^−1^), which is the phase transition.

For the CO:GT:PC mixture, both pre- and post oxidation compression isotherms showed a consistent small shift in the molecular area with each subsequent cycle at 10 mN m^−1^. Above the GT envelope transition pressure, the isotherms are very consistent for the unoxidized film, but show a significant decrease in reversibility for the oxidized films, likely because the GT envelope transition was inhibited due to oxidation (as observed in the BAM images). The CO:GT:PC hysteresis loop is again enlarged post-oxidation, albeit less prominently than in the case of CO:PC. The CO:GT:FFA:PC mixture is less respreadable and reversible by nature, even pre-oxidation, which may be attributable to the existence of saturated chain FFAs, which are known to reduce the respreadability of the film, as opposed to films primarily composed of unsaturated acyl chain lipids [16,82,83,85,86,87]. This low respreadability persists post-oxidation, as does the hysteresis pattern.

### 3.3. Rheological Parameters of TFLL Model Membranes

**Figure 8:**
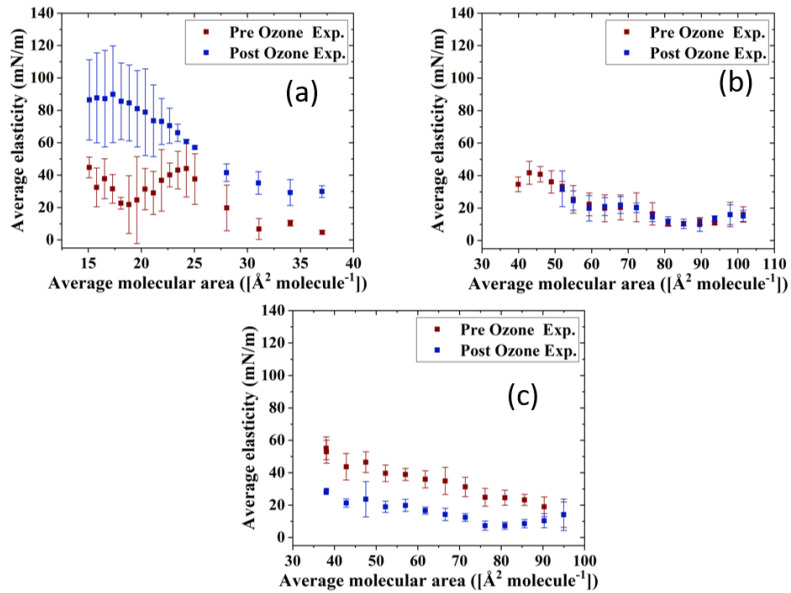
Surface dilational elasticity data of (**a**) CO:PC 90:10 (binary mixture); (**b**) CO:GT:PC 40:40:20 (ternary mixture); and (**c**) CO:GT:FFA:PC 40:25:15:20 (quaternary mixture) before ozone exposure (red) and after ozone exposure (blue) on PBS at 22 °C.

**Figure 9:**
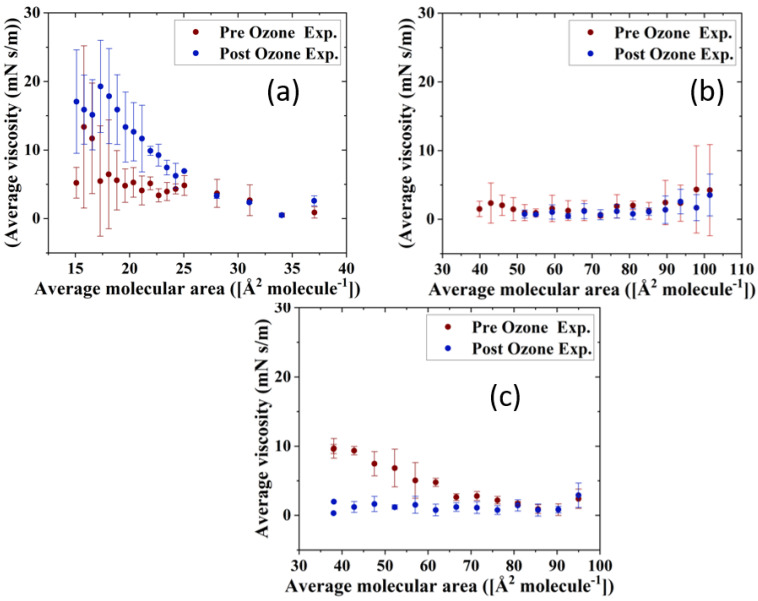
Surface dilational viscosity data of (**a**) CO:PC 90:10 (binary mixture); (**b**) CO:GT:PC 40:40:20 (ternary mixture); and (**c**) CO:GT:FFA:PC 40:25:15:20 (quaternary mixture) before ozone exposure (red) and after ozone exposure (blue) on PBS at 22 °C.

The impact of ozone exposure on the viscoelastic properties of the TFLL model membrane films is shown in Figure 8 and Figure 9. It can immediately be observed that measurements for the CO:PC binary mixture show much higher standard deviations compared with the other two mixtures, both for films pre- and post exposure to ozone. This is especially evident at molecular areas smaller than 27 Å^2^ molecule^−1^, areas at which the film shows evidence of structured multilayering. The inhomogeneity generated by the multilayering appears to affect the response of the film to oscillations, which is amplified with increasing hysteresis. Although the ozone exposure reduced the multilayering to smaller, discrete domains at higher surface pressures, the significant expansion in the isotherm means the multilayering occurs similar molecular areas both pre- and post-oxidation, and has a similar impact on the measurements. Moreover, the ozone exposure seems to have increased the dilational elasticity of the film throughout the compression. Again, it is important to remember that in the oxidized binary film, for the same areas, the surface pressure is much higher, which is reflected in the higher dilational elasticity and viscosity observed. In the case of dilational viscosity, the differences due to ozone exposure only manifest themselves in areas smaller than 23 Å^2^ molecule^−1^. The dilational viscosity and the elasticity increases can be attributed to the remaining CO moiety after the chain has cleaved off, adding to the dilational elasticity and viscosity of the film at areas where a condensed phase is formed by the remaining CO moiety.

Interestingly, the impact of ozone exposure on the ternary mixture is negligible for both the dilational elasticity and the dilational viscosity of the film. Compared to the binary mixture, the addition of GT has already reduced the multilayering and increased the fluidity of the pre-oxidation film, as seen in BAM (Figure 4). This dominance of the fluid phase is retained even after oxidation (Figure 4), which explains the low dilational viscosity and elasticity and the lack of change upon oxidation.

In contrast to both binary and ternary mixtures, ozone exposure decreases both the elasticity and the dilational viscosity of the quaternary mixture film, wherein the dilational viscosity of the film remains at ultra low values throughout the compression. It is important to note that unlike the binary mixture, for the quaternary mixture, the shift in molecular area upon oxidation is very small.

For the binary mixture, it was the change in the surface pressure at a given area that was a dominant factor in the dilational elasticity and viscosity increase upon oxidation. For the quaternary mixture, this was not the case; therefore, changes in the intermolecular interactions and morphology of the film are implicated. An intermediate molecular area shift is observed in the ternary mixture, as are morphology changes similar to those in the quaternary mixture. Thus, it can be deduced that the apparent small impact of ozone exposure on the ternary mixture is probably due to the existence of competing factors.

One such factor is clearly the reduced CO content in both the ternary and quaternary mixtures compared with the binary mixture. Thus, the oxidation products, which seemingly are the reason behind the increase in the binary mixture’s dilational elasticity and viscosity, are now present in lower amounts; their impact is observed in the form of no impact on or a reduction in the dilational elasticity and viscosity of the ternary and quaternary mixture films. It can also be deduced that GT’s oxidation products also have an increasing impact on the viscoelastic properties of the films, as can be seen from the lowered dilational elasticity and viscosity of the quaternary mixture post oxidation, as opposed to the negligible impact of oxidation on the ternary mixture.

## 4. Discussion

The purpose of this work was to study the impact of exposure to ozone on the mechanical properties of TFLL model membranes. Surface ozone oxidation generated aldehydes, carboxylic acids and in some cases, stable ozonide, hydroxy hydroperoxides and some Criegee intermediates. Ozone exposure leads to an increase in surface pressure as well as an expansion to higher molecular areas in the surface pressure-area isotherms of the TFLL model membranes, attributable to the accommodation of cleaved chains in the film which is most apparent for the binary mixture and less so for the ternary and the quaternary mixtures. This may be due to competing factors impacting the film’s surface behaviour. Moreover, the oxidation has a significant impact on the morphology of all of the TFLL model membranes, fluidizing the binary and ternary mixture films and disrupting the CO condensed phase domain growth and multilayering behaviour as well as the phase transition of GT. The oxidation of CO, GT, and PC leads to a more unstable film. The presence of FFAs, which do not themselves oxidize, impacts the line tension and leads to a higher condensed phase surface coverage post oxidation. The impact of ozone oxidation on the film respreadability is strongly correlated with GT content and alteration of the GT envelope transition, which has been shown to be reversible in unoxidized film, thereby adding to the stability of the film. The effect of oxidation on the film dilational elasticity and viscosity is highly dependent on film composition, underlining the importance of using appropriate model membranes.

Thus, it is evident that exposure to high concentrations of ozone induces significant changes in the surface characteristics and behaviour of TFLL model membranes, impacting their surface activity, morphology, viscoelasticity and stability, all of which are paramount in the proper function of the physiological TFLL. The results of this work help shed light on the impact of ozone on the TFLL and ocular surface health, on which little investigation has been carried out previously. This can aid in better understanding of the correlation between a compromised structure of the TFLL in DED patients and ozone concentrations in the troposphere layer, as well as prevention planning.

## Data Availability

All data were mentioned in this paper.

## Supplementary Materials

The following supporting information can be downloaded at: https://www.mdpi.com/article/10.3390/membranes13020165/s1, Section 1: rheology measurement program details; Figure S1: Criegee mechanism for ozonolysis of cholesteryl oleate; Figure S2: contrast, brightness and sharpness Enhanced BAM images of CO:PC 90:10 (binary mixture) and CO:GT:FFA:PC 40:25:15:20 (quaternary mixture) after ozone exposure; Figure S3: compression–expansion cycles of binary, ternary, and quaternary mixtures; Table S1: ESI-MS data for oxidized cholesteryl oleate; Table S2: ESI-MS data for oxidized glyceryl trioleate; Table S3: ESI-MS data for oxidized egg PC.
