# Supplementary material for: Impact of Pollutant Ozone on the Biophysical Properties of Tear Film Lipid Layer Model Membranes
Source: Membranes (Basel). 2023 Jan 28;13(2):165. doi: 10.3390/membranes13020165 (PMC9964828; doi:10.3390/membranes13020165)
Supplement: Supplementary file 1 [file membranes-13-00165-s001.zip › membranes-2147646-supplementary.pdf]

membranes

MDPI

Article

Impact of Pollutant Ozone on the Biophysical Properties of Tear Film Lipid Layer Model Membranes-Supplementary Information

Mahshid Keramatnejad [1], Christine DeWolf *

[1] Department of Chemistry & Biochemistry & Centre for Nanoscience Research, Concordia University,7141 Sherbrooke Street west, H4B1R6, Montreal, Canada; Mahshid.Keramatnejad@concordia.ca

* Correspondence: Christine.DeWolf@concordia.ca ; Tel.: +1-(514)-848-2424 (ext.)

1. Rheology Measurements Program Details

A system-specific program was built to specifically account for the changes of 1 mN min $^{-1}$ in the slope of the pressure-area isotherms of each film, allowing efficient experiment time. The CO:PC 90:10 (binary mixture) program underwent an initial compression from 40 mm^2 to 37 mm^2, after which there was an equilibration time of 300 seconds. The drop then underwent 25 oscillations for 156.25 s with an amplitude of 2.50 % (of the drop area) and a frequency of 0.16 Hz [64] followed by an equilibration time of 180 s, then compressed to 34 mm^2, equilibrated (300 s), and underwent oscillations of the same parameters. After 4 more repeats of this cycle, the program moved on to the next part where, following the binary mixture isotherm slope change, the drop area change was reduced to ensure the 1 mN min $^{-1}$ surface pressure increase. Thus, after the 5th. oscillations cycles and the equilibration time (180 s), the drop area was reduced from 25 mm^2 to 24.2 mm^2. The same cycle was repeated until the drop area of 15.1 mm^2 was reached with the rheology experiments providing the measurements of viscosity and elasticity of the film [57,58,60].

The initial compression of the CO:GT:PC 40:40:20 (ternary mixture) film was from 40 mm^2 to 38.66 mm^2, after which the drop was equilibrated (300 s) and oscillated with the same parameters as before. After the second set of oscillations and the following equilibration time (180 s), to ensure the 1 mN min $^{-1}$ increase, the amount of reduction of the drop area was changed. The drop area was reduced from 37.33 mm^2 to 35.69 mm^2, followed by equilibration (300 s), oscillations with the same parameters as before, equilibration (180 s) and the next compression. This cycle was repeated until the 12$^{th.}$ set of oscillations had been completed, after whose equilibration (180 s), the next compression comprised of drop area reducing from 20.95 mm^2 to 19.8 mm^2. The same cycle was repeated for a total of 5 times until a drop area of 15.23 mm^2 was achieved.

The initial compression of the CO:GT:FFA:PC 40:25:15:20 (quaternary mixture) program changed the drop area from 40 mm^2 to 38 mm^{2,} followed by an equilibration time of 300 s, oscillations with similar parameters as before, and an equilibration time of 180 s. The program then entered its second stage where a change in the drop area reduction size was needed, thus the drop area was reduced from 38 mm^2 to 36.1 mm^2, followed by equilibration time (300 s), oscillations of the same parameters and equilibration time (180 s). This cycle was repeated for a total of 12 times, until the area of the drop reached 15.2 mm^2, completing the rheology measurements and providing the dilational surface elasticity and dilational viscosity of the film. For each system, the experiment was repeated at least 4 separate times.

2. Criegee Mechanism of Ozonolysis of Cholesteryl Oleate

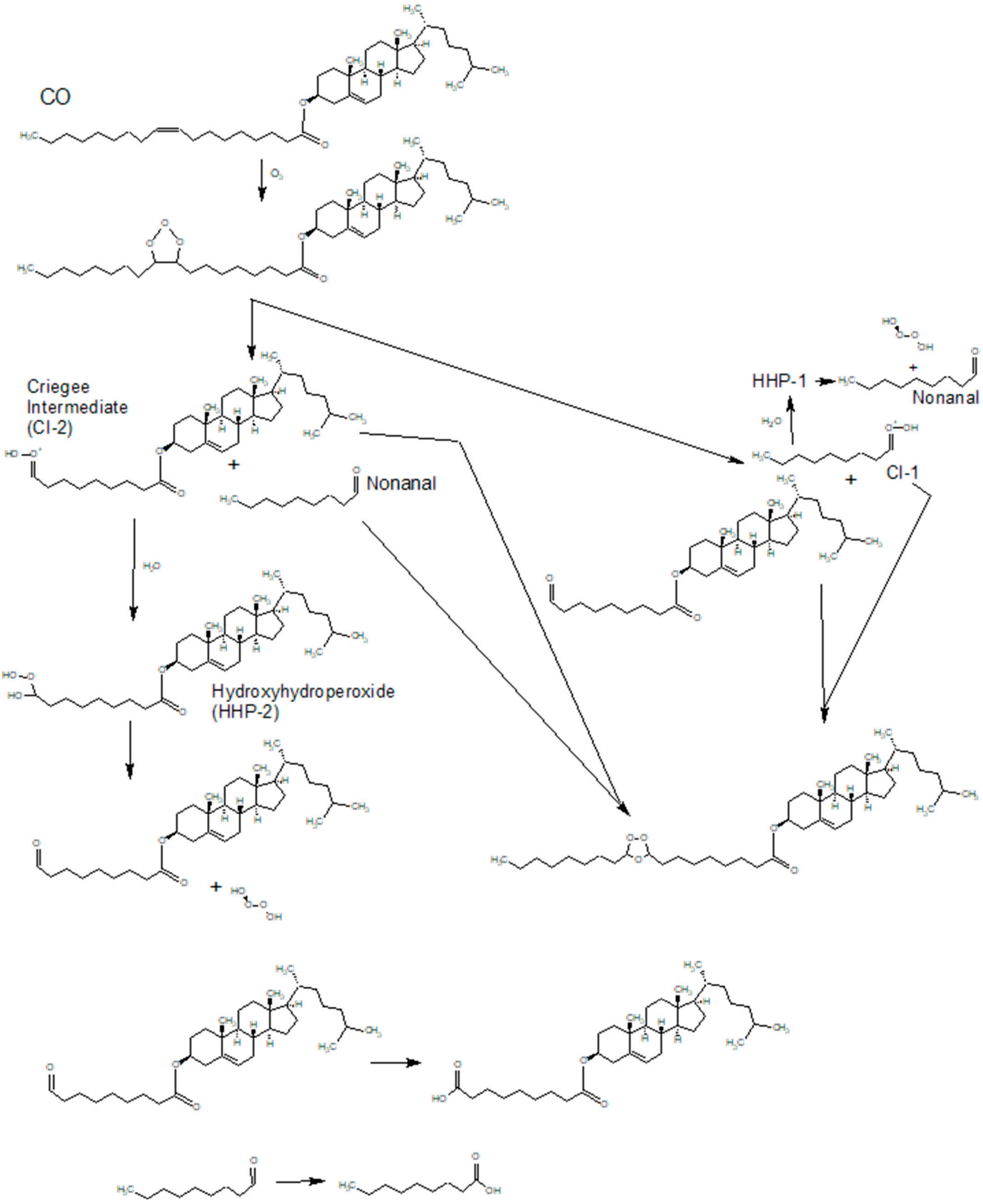

Figure S1. Details of the ozonolysis mechanism for cholesteryl oleate (CO) via the Criegee intermediate.

3. Contrast, Brightness & Sharpness Enhanced BAM Images of CO:PC 90:10 (Binary Mixture) and CO:GT:FFA:PC 40:25:15:20 (Quaternary mixture) After Ozone Exposure

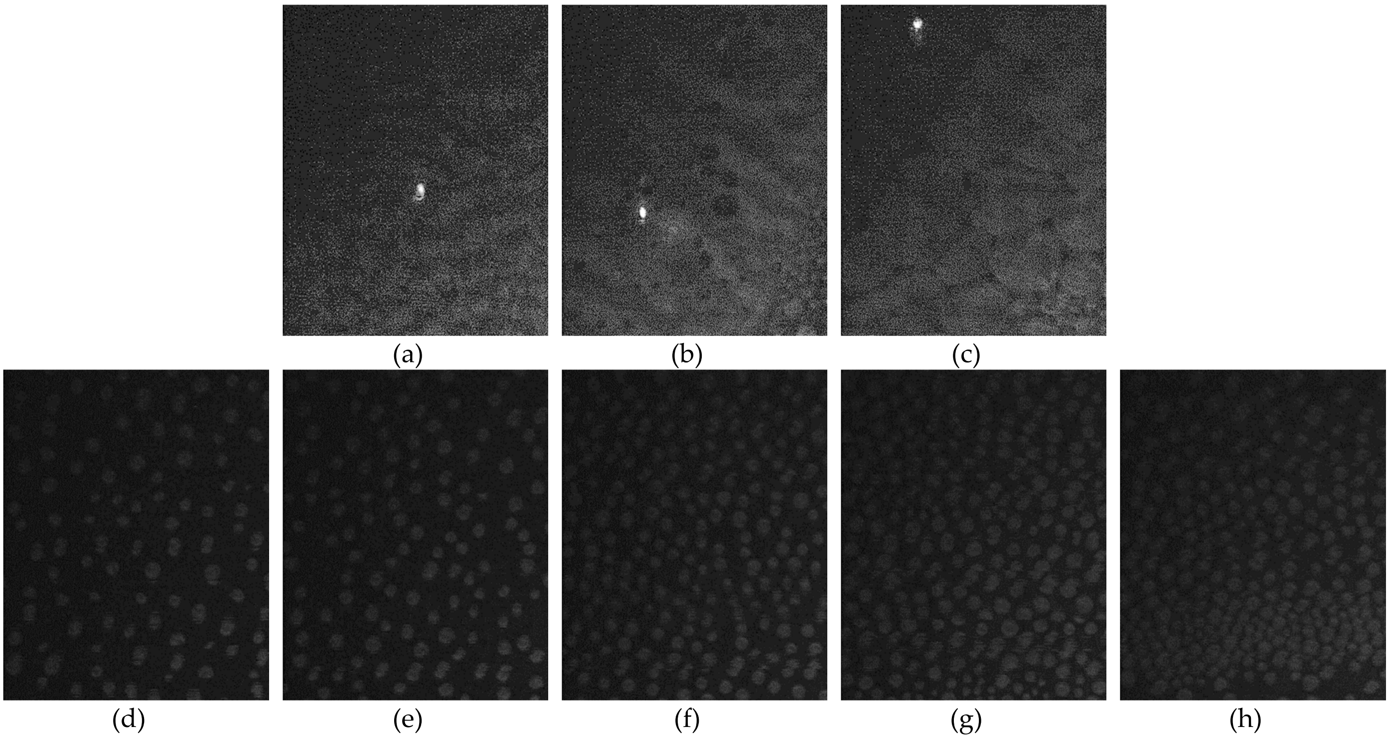

Figure S2. Contrast, brightness and sharpness enhanced BAM images (220 μm wide) of TFLL model membranes after ozone exposure on PBS at 22 °C . TOP ROW: CO:PC 90:10 (binary mixture) film at surface pressures (a) 3 mNm^{-1}; (b) 4 mNm^{-1}; and (c) 5 mNm^{-1}. The enhanced BAM images of the film at these surface pressure show the progression of the appearance of circular domains with low contrast. BOTTOM ROW: CO:GT:FFA:PC 40:25:15:20 (quaternary mixture) at surface pressures: (d) :5 mNm^{-1}; (e): 9 mNm^{-1}; (f): 13 mNm^{-1} (g): 16 mNm^{-1}; and (h): 18 mNm^{-1}.

4. Compression-Expansion Cycles of Binary, Ternary, & Quaternary Mixtures

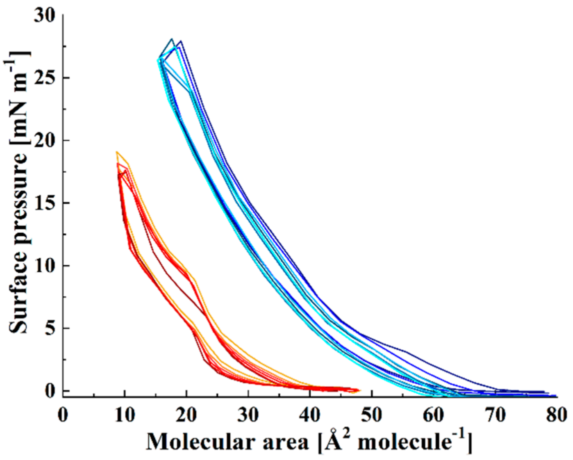

(**a**)

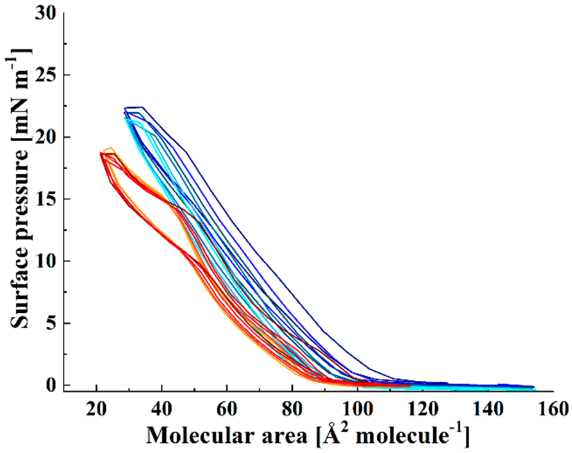

(**b**)

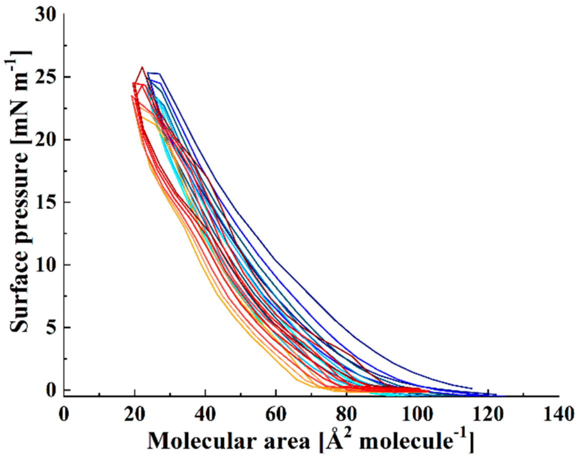

(**c**)

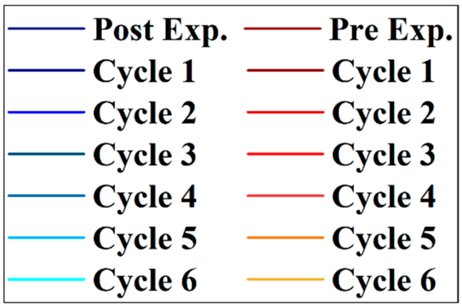

(**d**)

Figure S3. Compression-expansion cycles of (a): CO:PC 90:10 (binary mixture); (b): CO:GT:PC 40:40:20 (ternary mixture); (c): and CO:GT:FFA:PC 40:25:15:20 (quaternary mixture) before ozone exposure (d,red color family) and after ozone exposure (d,blue color family) on PBS at 22 °C.

5. Products of Ozonolysis

Table S1. ESI-MS data for oxidized cholesteryl oleate.

Structure	Theoretical m/z	Observed m/z	Mass Error (ppm)	Signal Intensity	Mode
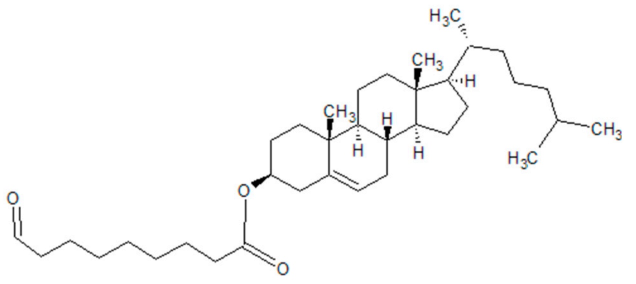	541.4615	541.4614	-0.0907	1.13E+05	Positive
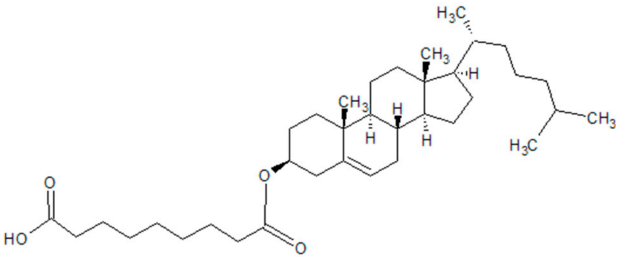	557.4564	557.4566	0.3606	8.94E+04	Positive
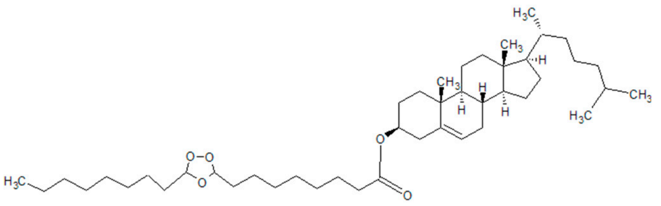	699.5922	699.5957	5.0825	2.72E+06	Positive

Table S2. ESI-MS data for oxidized glyceryl trioleate.

Structure	Theoretical m/z	Observed m/z	Mass Error (ppm)	Signal Intensity	Mode
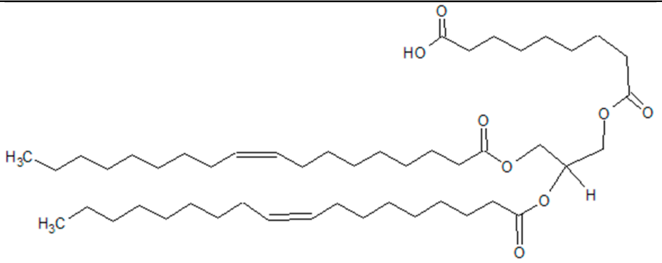	789.6249	789.6239	-1.3773	7.74E+03	Negative
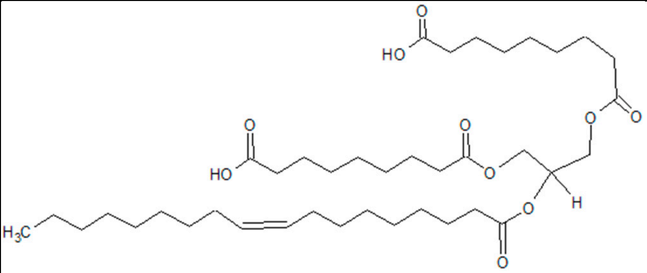	695.4739	695.4738	-0.1847	4.93E+03	Negative
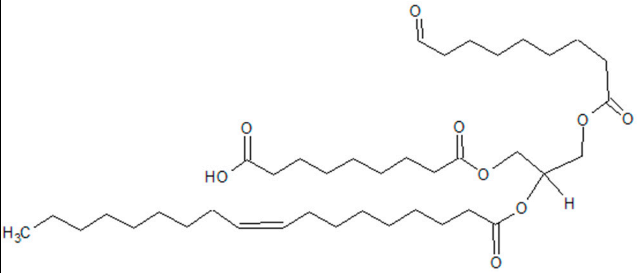	679.4790	679.4793	0.4644	1.18E+04	Negative
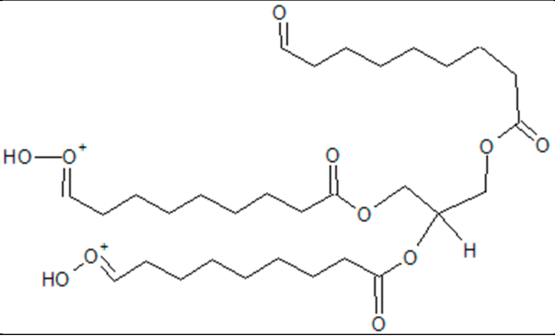	585.3280	585.3281	0.2577	1.28E+06	Negative

Structure	Theoretical m/z	Observed m/z	Mass Error (ppm)	Signal Intensity	Mode
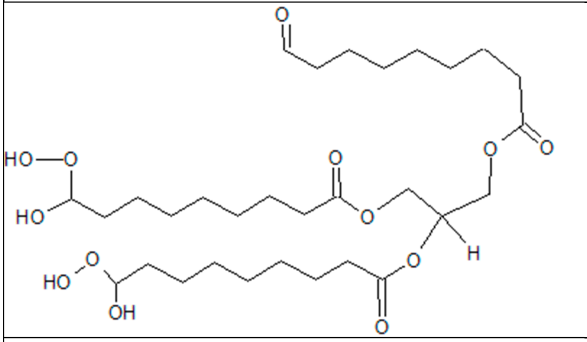	621.3491	621.3412	-12.6749	1.44E+05	Negative
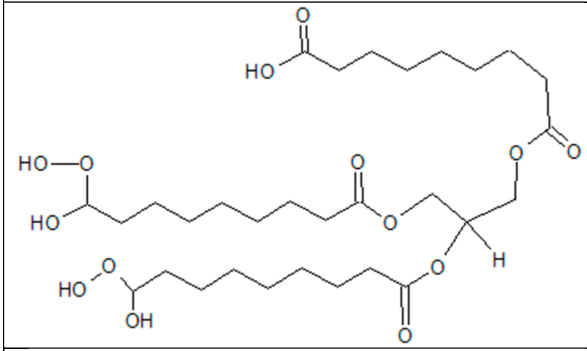	637.3440	637.3483	6.6855	4.91E+03	Negative
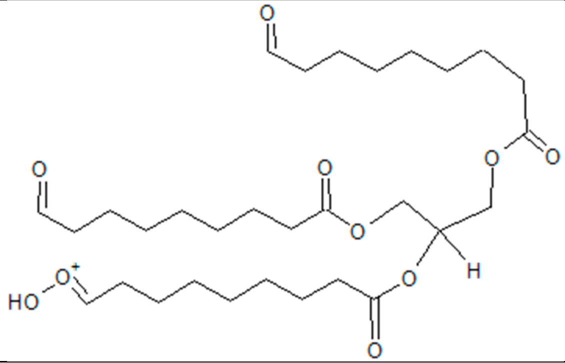	569.3331	569.3333	0.3751	1.37E+06	Negative
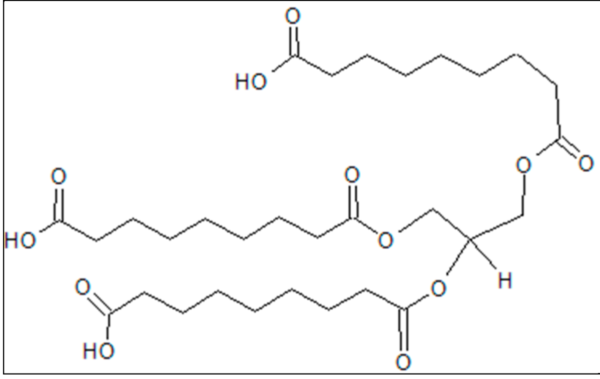	601.3229	601.3231	0.4067	1.38E+05	Negative

Table S3. ESI-MS data for oxidized egg PC.

Structure	Theoretical m/z	Observed m/z	Mass Error (ppm)	Signal Intensity	Mode
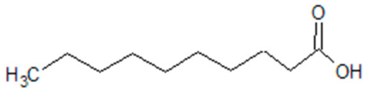	171.1390	171.1393	1.5435	1.06E+06	Negative
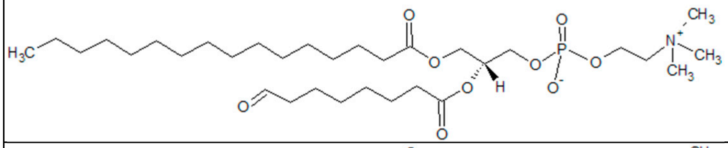	635.4167	635.4115	-8.2534	4.09E+03	Negative
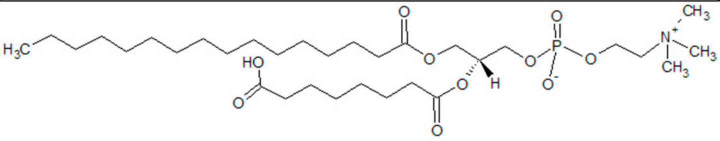	651.4116	651.4111	-0.8842	5.67E+03	Negative
